# 50 Years of Cumulative Open-Source Data Confirm Stable and Robust Biodiversity Distribution Patterns for Macrofungi

**DOI:** 10.3390/jof8090981

**Published:** 2022-09-19

**Authors:** Haili Yu, Tiejun Wang, Andrew Skidmore, Marco Heurich, Claus Bässler

**Affiliations:** 1 Faculty of Geo-Information Science and Earth Observation, University of Twente, 7514 AE Enschede, The Netherlands; 2 Department of Earth and Environmental Science, Macquarie University, Sydney 2109, Australia; 3 Chair of Wildlife Ecology and Wildlife Management, University of Freiburg, 79105 Freiburg, Germany; 4 Bavarian Forest National Park, 94481 Grafenau, Germany; 5 Institute for Forest and Wildlife Management, Inland Norway University of Applied Science, 2480 Koppang, Norway; 6 Institute for Ecology, Evolution and Diversity, Faculty of Biological Sciences, Goethe University Frankfurt, 60323 Frankfurt, Germany

**Keywords:** species distribution model, species discovery curve, species richness, hotspot

## Abstract

Fungi are a hyper-diverse kingdom that contributes significantly to the regulation of the global carbon and nutrient cycle. However, our understanding of the distribution of fungal diversity is often hindered by a lack of data, especially on a large spatial scale. Open biodiversity data may provide a solution, but concerns about the potential spatial and temporal bias in species occurrence data arising from different observers and sampling protocols challenge their utility. The theory of species accumulation curves predicts that the cumulative number of species reaches an asymptote when the sampling effort is sufficiently large. Thus, we hypothesize that open biodiversity data could be used to reveal large-scale macrofungal diversity patterns if these datasets are accumulated long enough. Here, we tested our hypothesis with 50 years of macrofungal occurrence records in Norway and Sweden that were downloaded from the Global Biodiversity Information Facility (GBIF). We first grouped the data into five temporal subsamples with different cumulative sampling efforts (i.e., accumulation of data for 10, 20, 30, 40 and 50 years). We then predicted the macrofungal diversity and distribution at each subsample using the maximum entropy (MaxEnt) species distribution model. The results revealed that the cumulative number of macrofungal species stabilized into distinct distribution patterns with localized hotspots of predicted macrofungal diversity with sampling efforts greater than approximately 30 years. Our research demonstrates the utility and importance of the long-term accumulated open biodiversity data in studying macrofungal diversity and distribution at the national level.

## 1. Introduction

Fungi are a hyper-diverse kingdom that contributes significantly to the regulation of the global carbon and nutrient cycle [1,2,3]. Studying fungal diversity and distribution patterns is essential in shedding light on the functioning of terrestrial ecosystems [4,5,6]. However, compared to plants and animals, our understanding of the diversity and distribution patterns of fungi at a large spatial extent, e.g., a national or continental scale, remains limited [7,8]. One of the main reasons for this is the challenge of sampling, as most fungi live underground or inside substrates and are hard to observe directly in the field [9,10,11]. Nevertheless, there is a large group of fungal species that produce visible fruiting bodies (hereafter macrofungi), which are useful for recording species presence and studying the diversity and distribution of fungi in a reliable manner [7,12,13,14,15].

However, the macrofungal occurrence is also challenging to detect and predict due to their cryptic characteristics. The fruiting of macrofungi is primarily determined by climate and can vary considerably depending on related environmental factors at different geographic locations from year to year [16], which requires long-term sampling. Based on cultivation experiments of different macrofungal species in different regions, some studies found that the fungal fruit bodies seem to appear only when the moisture of soil and atmosphere is near saturation, such as after a rain event [17,18,19]. However, no universal set of conditions leads to fungal species fructification [17]. In addition, the emergence of fruiting bodies of many fungal species can be ephemeral, and their lifespan is species-specific that varies from a few days to weeks [14,20]; hence, the detection of macrofungal diversity and distributions requires intensive field sampling [13]. Many studies have demonstrated that macrofungi require a high sampling frequency and a long investigation period. For example, by randomly investigating several Swiss forests, Egli et al. [21] found that reduced sampling frequency in the field can cause a loss of recorded species. Straatsma and Krisai-Greilhuber [22] reported that half of the species were observed only once during a seven-year survey of fungal fruit bodies in 13 forests and grassland plots near Vienna, indicating a large annual variation in macrofungal species richness. In another study, Straatsma et al. [14] found that only 8 out of 408 species were observed in all years in a 21-year survey on fungal fruit bodies in the fungal reserve La Chaneaz in western Switzerland. As such, the macrofungal survey conducted only once, or even over several years, is likely to underestimate the macrofungal species diversity. Therefore, long-term investigation and sample collection are crucial to studying macrofungal diversity and distribution. However, long-term field collection (e.g., 10 years or more) and high-frequency monitoring are time consuming and labor intensive, making studying macrofungal diversity and distribution extremely challenging, especially on a large spatial scale.

Natural history collections over hundreds of years offer extensive species distribution information with considerable coverage. By connecting multiple collections from different regions, these databases can cover a broad variety of taxonomic groups at regional and even global coverage [23], which is almost impossible to achieve for well-designed systematic field surveys [23,24,25]. In recent years, these valuable species occurrence data have been increasingly digitized in electronic databases and shared online, such as the Global Biodiversity Information Facility (GBIF, http://www.gbif.org/) [26,27,28]. To date, the GBIF data portal provides free access to more than two billion species occurrence records. This growing number of digitized and georeferenced species occurrence records has created an opportunity to monitor species diversity and distribution patterns at large spatial scales over extended time periods [29,30]. However, there are major concerns about the use of such datasets, as the scientific community acknowledges that these species occurrence records result from years of different researchers working with different aims and methodologies are often biased due to variation in sampling efforts in space and time [27,31,32]. These sampling efforts are scattered and often seriously biased toward easily accessible areas, such as close to the road or near the town [33,34,35]. Furthermore, such biases can lead to deriving erroneous associations of species with environmental variables, inferring incorrect species’ absences [36,37]. These concerns have challenged the utility of open biodiversity data in revealing species diversity and distribution patterns. Nevertheless, open biodiversity data have advanced our understanding of species diversity and distribution and informed conservation policy for a wide range of species [38]. For example, based on compiled open-access global marine species distribution data, Tittensor et al. [39] studied the global marine biodiversity pattern and indicated the fundamental role of temperature in structuring marine biodiversity. Using occurrence data of terrestrial vertebrates obtained from GBIF, Mayani-Parás et al. [40] projected terrestrial vertebrates distribution and quantified their spatiotemporal habitat loss.

**Figure 1:**
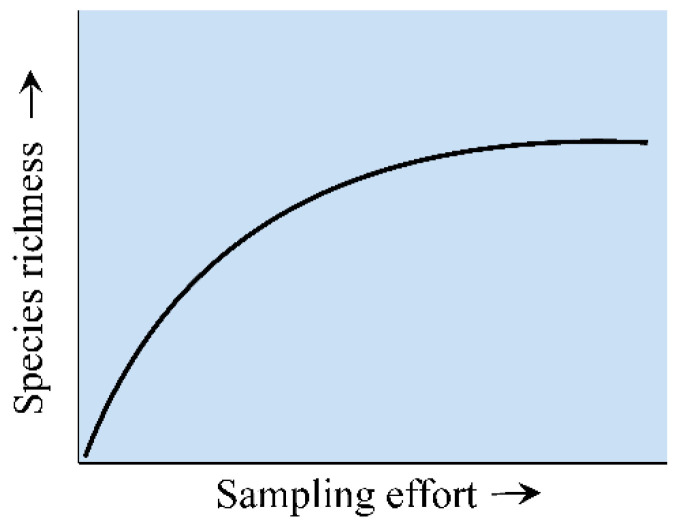
A conceptual diagram of species accumulation curves showing the cumulative number of species (species richness) recorded in a particular area as a function of the cumulative effort expended searching for them (sampling effort).

The theory of species accumulation curves (also called species–effort curves or species discovery curves) predicts that the cumulative number of species found in one particular area would increase with the cumulative sampling effort (e.g., increasing individuals, samples size, sample time), and it will tend to flatten when the sampling effort (e.g., time spent in collecting observations) is sufficiently large [41,42,43,44,45]. A conceptual diagram of species accumulation curves is presented in Figure 1. Therefore, datasets with the accumulated species number observed trending to reach the asymptote with increasing sampling effort might also represent relative completeness of inventory.

The GBIF contains a large amount of macrofungal occurrence data, which have been collected by amateur mycologists, researchers, and naturalists for centuries, especially rich in Europe than in any other region [7,46]. Such long-term accumulation of sampling raises the question of how the decades’ data accumulate species and whether these data could be used to reveal the diversity and distribution patterns of macrofungal species on a national scale. In recent years, there has been an increase in macrofungal studies using a variety of open-source biodiversity data [15,46,47,48,49,50]. Yet, studies focusing on macrofungal diversity and distribution at a large spatial scale are still limited [15,47].

Norway and Sweden both have a long tradition of mycological studies and are among the regions where the fungi have been most thoroughly investigated [15,51]. Using long-term accumulated macrofungal species occurrence data in Norway and Sweden (and downloaded from the GBIF portal) as cases, we performed species distribution models at different sampling periods and aimed to examine changes in macrofungal diversity and distribution patterns at the national scale. We hypothesized that there should be an increase in the breadth of spatial data coverage over time, and the cumulative number of recorded species could flatten with data accumulation. Thus, these data might represent a relatively comprehensive sample, and with the application of the species distribution model, these data should contain the potential to predict macrofungal diversity and distribution patterns across the country. To test this hypothesis, we sought to determine (1) Does the cumulative number of macrofungal species reach an asymptote with increasing sampling effort? and (2) Can the long-term cumulative sampling of GBIF be used to confirm stable and robust biodiversity distribution patterns for macrofungi at the national scale?

## 2. Materials and Methods

### 2.1. Occurrence Data of Macrofungal Species

We downloaded georeferenced fungal occurrence records collected between 1970 and 2020 in two European countries (i.e., Norway and Sweden) from the Global Biodiversity Information Facility (GBIF; http://data.gbif.org, accessed on 17 February 2022). As we only study macrofungal species, we focused on species in the class Agaricomycetes. The class Agaricomycetes, which is under the phylum Basidiomycota, is one of the largest and most conspicuous groups of fungi, and is comprised of both striking diverse morphology of fruiting bodies and nutritional modes [11,52]. In order to further assure the macrofungal species, we filtered species records using the checklist derived from the macrofungal study in Europe by Sánchez-García et al. [52] and Andrew et al. [53]. We then placed all macrofungal occurrence data in 5 km × 5 km grid cells, which were generated based on European Terrestrial Reference System 1989 (ETRS89) data and Lambert Azimuthal Equal 176 Area (LAEA) projection (EPSG: 3035) across Norway (14,056 grids) and Sweden (17,597 grids). To improve the consistency of the macrofungal data, records flagged with taxonomic problems by GBIF (i.e., records flagged with “taxon_match_fuzzy” and “taxon_match_higherrank” issue) and those without specific species names were deleted. Finally, we excluded the records with a “coordinate uncertainty” larger than 5 km.

**Figure 2:**
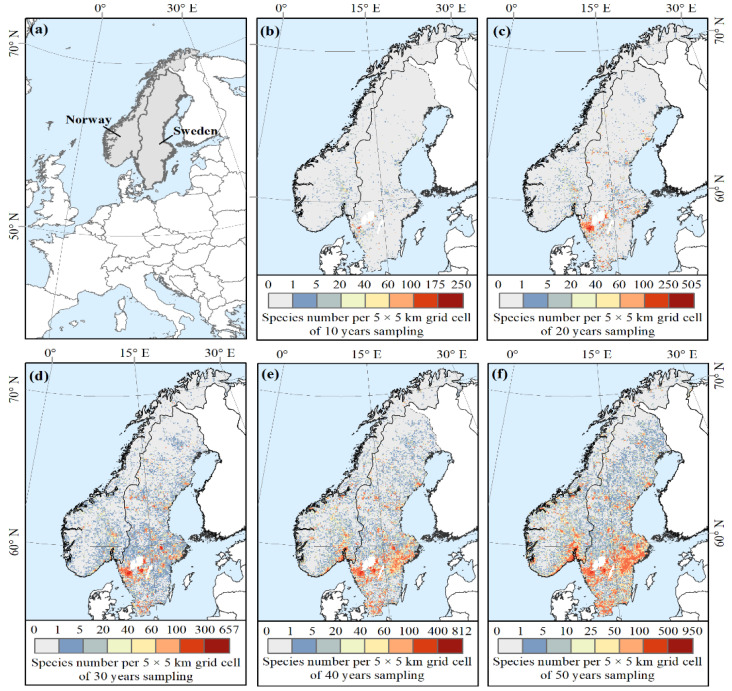
Location of the study area (**a**) and the distribution of the number of macrofungal species per grid cell used in this study for (**b**) 10 years (1970–1980), (**c**) 20 years (1970–1990), (**d**) 30 years (1970–2000), (**e**) 40 years (1970–2010), and (**f**) 50 years (1970–2020) sampling.

We divided the filtered macrofungal data into five groups with different sampling efforts for each country: 10 years (1970–1980), 20 years (1970–1990), 30 years (1970–2000), 40 years (1970–2010), and 50 years (1970–2020). For each group in each country, we further removed species that occupy less than 30 grid cells to ensure a robust model performance and ensure access to a reasonable number of test data for modelling [54]. Finally, we removed the duplicated records of each species in each 5 km × 5 km grid cell to reduce occurrence localities correlation. As a result, we compiled two databases with 1168 and 1422 macrofungal species in Norway and Sweden, respectively, for use in our model (Table 1 and Figure 2).

To test whether the cumulative number of macrofungal species reaches an asymptote when the sampling effort is sufficiently large, we analyzed the data by fitting an asymptotic regression model [55] using the following formula:

(1) $$y=a-bc^{x}$$

where *y* is the cumulative number of species, *x* is the year of sampling, *a* represents the asymptote of the curve, *b* is the difference between the value of *y* (when *x*  =  0) and the asymptote, and *c* is the rate constant at which the maximum is reached.

### 2.2. Predictor Variables

Based on prior knowledge of macrofungal growth and their diversity and distribution at a large spatial scale, we selected climatic variables, tree species and soil-related variables for predicting the distribution of macrofungi. Given that macrofungal reproduction, distribution, and physiology are largely influenced by annual temperature and precipitation [46,56], and the timing of macrofungi fruiting has been found to be sensitive to seasonal climate [16,46,57], we selected both annual and seasonal climatic factors that are essential to macrofungal species based on prior knowledge [16,58,59]. Therefore, we downloaded both annual and seasonal bioclimatic variables for species distribution modelling (Table 2) at 2.5′ resolution (~5 km) from WorldClim (https://www.worldclim.org/, accessed on 19 October 2021). These bioclimatic variables are derived from monthly temperature and precipitation between 1970 and 2000 [60,61], and are widely used in ecological studies.

In addition, tree species identify and tree species diversity are found largely associated with macrofungal species richness, especially for the ectomycorrhizal fungal species and at the large spatial scale [46,47,50]. Therefore, we considered the two most important vegetation-related variables, i.e., dominant tree species and tree species diversity, in the species distribution model. We downloaded the distribution maps at a resolution of 1 km × 1 km of 20 dominant tree species over Europe from the European Forest Institute (EFI Joensuu, Finland; https://www.efi.int/knowledge/maps/treespecies, accessed on 3 June 2021). Based on these tree distribution maps [62], we calculated dominant tree species and tree species diversity in each grid cell.

Furthermore, soil condition, such as soil organic carbon, soil pH and bulk density, is also widely found to highly correlate with fungal growth and distribution [50,59,63]. Thus, we downloaded the related data of these three soil characteristics from SoilGrid (https://files.isric.org/soilgrids/latest/data/, accessed on 2 September 2022). All variables used were listed in Table 2.

### 2.3. Species Distribution Modelling

We used MaxEnt (version 3.4.3) to model the distribution of each species in each sampling period in each country. MaxEnt is a presence-only species distribution model, allowing scientists to make use of rich data sources from natural history collections [58]. The MaxEnt model can perform well with correlated variables by using regularization, which is a common approach to model selection, as well as an inbuilt method that deals with feature selection by relegating some coefficients to zero to avoid overfitting, so there is less need to remove correlated variables [64,65]. Furthermore, since the purpose of the study was not to examine the importance of variables, we included all 19 bioclimatic variables as environmental variables in our model.

As our macrofungal occurrence data are presence-only data, we need to generate pseudo-absence data across the study area for each species to substitute true absences in MaxEnt. These pseudo-absence data, usually randomly distributed, contrast against the presence locations and represent the range of environmental conditions [64,66]. However, the species occurrence records from open-source platforms are often thought to be spatially biased (e.g., toward easily accessible areas), which may lead to inaccurate inferences and predicted distributions [36]. Thus, instead of randomly generating the pseudo-absence points, we used a bias file, an option implemented in the MaxEnt software, to assign weight to select pseudo-absence points for macrofungal species. For each country in each group, we produced bias grids by deriving a Gaussian kernel density map [67,68] of all macrofungal species occurrence locations with the “kde2d” function of the “MASS” package [69] in R version 4.0.3 [70]. The selection of pseudo-absence points reflects the same bias as the presence data and can ameliorate the effects of sampling bias of presence data [37,71]. We sampled 10,000 pseudo-absence points for each species according to the bias surface. The default parameters in the MaxEnt model were maintained, because previous simulations indicated that the default parameters could be as good as the fit parameters [72]. We performed 10–fold cross-validation and evaluated the model performance using the area under the receiver–operating characteristic curve (AUC) and the true skill statistics (TSS) [73]. Both AUC and TSS are the measures widely used to evaluate model performance in species distribution modelling [65,73]. AUC is a threshold-independent method, ranging from 0 to 1, where a score of 1 indicates perfect discrimination [74]. Whereas, the TSS is a frequently used threshold-dependent method that ranges from −1 to +1, where +1 indicates perfect agreement and values of zero or less indicate a model that performs no better than random, with a value greater than 0.5 widely used to indicate good model performance [75,76].

### 2.4. Calculation of Macrofungal Species Richness and Hotspots

The default output of the MaxEnt is a raster map with continuous values between 0 and 1, indicating the habitat suitability for a species. To generate the macrofungal species richness map, we firstly transformed the default output of the MaxEnt of each species into binary (presence/absence) predictions, using the maximum of the sum of sensitivity and specificity (Max SSS) as a threshold [77]. We then summed up the binary outcome to yield macrofungal species richness in each sampling period. To quantify the difference in the distribution pattern of macrofungal species richness generated in each time period, we calculated the Structural Similarity Index (SSIM) between each two adjacent species richness maps. The SSIM index is a spatial comparison method developed to determine the quality of image compression [78], which can be used to extract diversity and distribution pattern information from spatial ecological data [79]. As the SSIM performs better in the local area, we split each macrofungal richness map into 7 × 7 pixels using the Split Raster tool in ArcGIS 10.8.1. We calculated SSIM of the corresponding local maps of the two adjacent time periods using the “SSIM” function of the “SpatialPack” package [80] in R version 4.0.3. We then calculated the mean value of all local maps to represent the similarity of patterns on the national scale. The SSIM value ranges from 0 to 1, with a higher value indicating a higher similarity between maps [78]. To further explore the diversity and distribution pattern, we also calculated the diversity hotspot of macrofungal species based on the top 1–25% grid cells ranking by species richness [81].

## 3. Results

### 3.1. Accumulation of Macrofungal Species over Time

**Figure 3:**
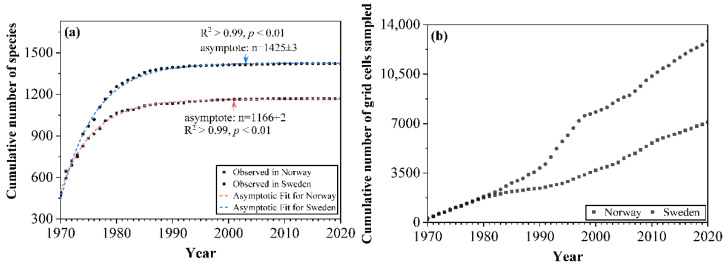
The cumulative number of (**a**) macrofungal species and (**b**) grid cells sampled (i.e., grid cell number with species occurrence inside) used in the model between 1970 and 2020 in Norway and Sweden.

**Figure 4:**
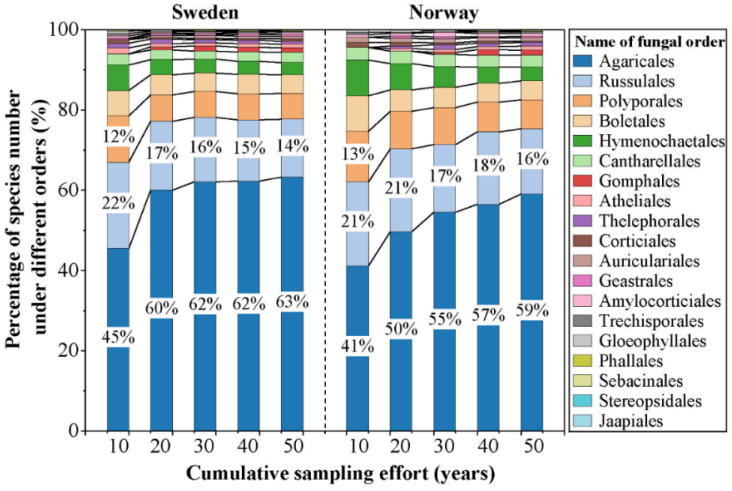
Percentage of species number under different orders in each accumulated sampling time (i.e., sampling for 10 years (1970–1980), 20 years (1970–1990), 30 years (1970–2000), 40 years (1970–2010), and 50 years (1970–2020)) of Norway and Sweden used in this study. The labels of a percentage less than 10% are not shown.

The cumulative number of macrofungal species in each country increased significantly with the accumulation of sampling time during the first 20 years (1970–1990), while the increase from 1990 onwards was relatively small, and few new species were added (Figure 3a). The asymptotic regression analysis showed that the cumulative number of species in both countries reached the asymptote (R^2^ > 0.99; *p* < 0.01) after more than 30 years’ accumulation of data (Figure 3a). The cumulative number of grid cells sampled (i.e., grid cells with macrofungal species occurrence inside) showed an increasing trend with the accumulation of sampling time for each country (Figure 3b), representing the increase of spatial data coverage. For the species composition, with the increased sampling time, fewer species under new orders were added; the species compositions by orders became more and more similar with a preponderance of the species within the order of the Agaricales (Figure 4).

### 3.2. Model Performance

**Figure 5:**
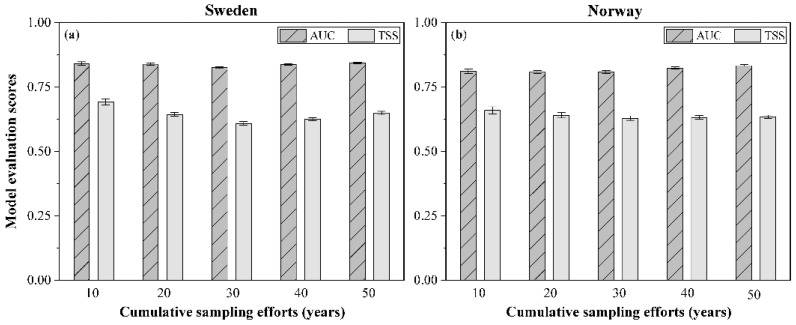
Mean value of the area under the receiver–operating characteristic curve (AUC) and the true skill statistic (TSS) for all species in each cumulative sampling period for (**a**) Sweden and (**b**) Norway. Both AUC and TSS were used to evaluate the model performance for each individual species.

Overall, the models for species in each cumulative sampling period for Sweden and Norway performed well, with a mean AUC ranging from 0.81 to 0.84 and a mean TSS ranging from 0.63 to 0.66 (Figure 5). The standard deviation depicted on each bar in Figure 5 shows the variation of model performance among species.

### 3.3. Predicted Macrofungal Species Richness and Diversity Hotspots

**Figure 6:**
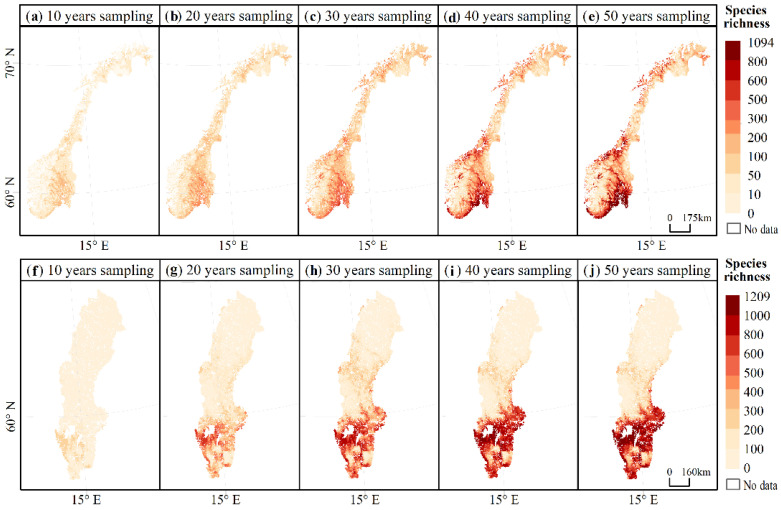
Modelled distribution of macrofungal species richness under different accumulated sampling times (i.e., sampling for 10 years (1970–1980), 20 years (1970–1990), 30 years (1970–2000), 40 years (1970–2010), and 50 years (1970–2020)) of (**a**–**e**) Norway and (**f**–**j**) Sweden.

**Figure 7:**
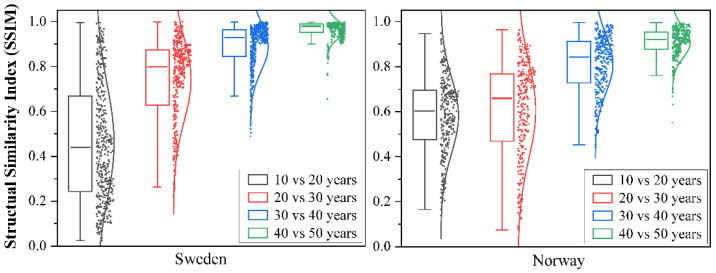
Box plot of the Structural Similarity Index (SSIM) value calculated between the two species richness maps from each of two adjacent sampling periods (i.e., between 20 and 10 years; between 30 and 20 years; between 40 and 30 years; and between 50 and 40 years) for Sweden and Norway. The thick black line denotes the median value, and the points next to the box plots represent the SSIM value of split local maps.

Figure 6 shows the predicted macrofungal species richness for Norway and Sweden over five accumulative sampling periods. The similarity between the two species richness maps predicted for adjacent sampling periods was quantified using the structural similarity index (SSIM), as illustrated in Figure 7. At the beginning 20 years, the overall SSIM is lower with high variation among patches. After the accumulation of macrofungal data for longer than 30 years, the SSIM of the predicted macrofungal species richness pattern increased significantly to a high value of around 0.9, indicating a significantly high similarity of the macrofungal distribution pattern. Additionally, there is a much smaller variation of the SSIM values among patches across the two counties (Figure 7).

**Figure 8:**
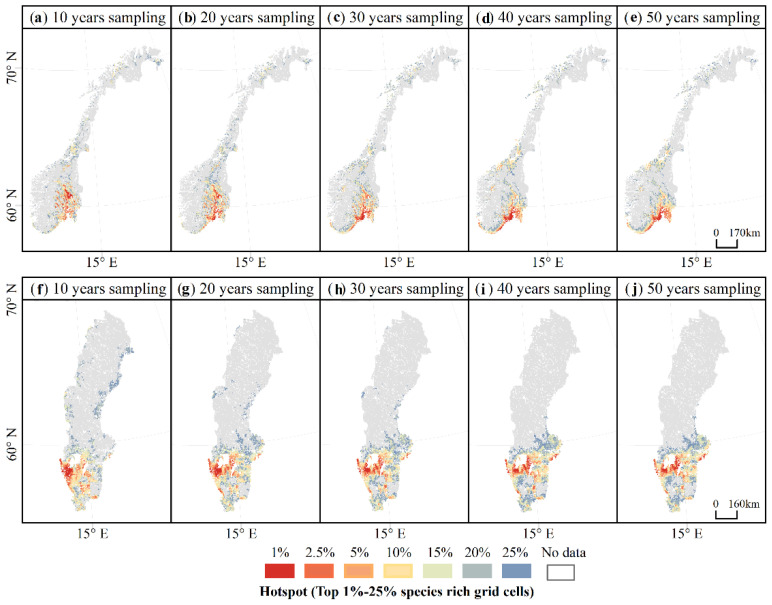
The distribution of the hotspots of macrofungal species richness predicted in each accumulated sampling period (i.e., sampling for 10 years (1970–1980), 20 years (1970–1990), 30 years (1970–2000), 40 years (1970–2010), and 50 years (1970–2020)) for (**a**–**e**) Norway and (**f**–**j**) Sweden.

There were also increasing similarities in the distribution of the hotspots of macrofungal species richness, especially for the hotspots of the top 1% and 2.5% species-rich grid cells as the length of sampling increased above 30–40 years. For Norway, after accumulating above 40 years of data, the predicted macrofungal hotspot areas concentrate in the south part and are mainly around 60° N in the far southeast of the country (Figure 8a–e). For Sweden, after accumulating 30 years of data, the predicted macrofungal hotspot areas were distributed in a band around 58–60° N (Figure 8f–j).

## 4. Discussion

By exploring 50 years of open macrofungal data in Sweden and Norway, our study showed that the rate of species accumulation decreased with the accumulation of sampling effort. As the collection builds over time, the predicted distribution patterns of macrofungal species richness and diversity hotspots became increasingly similar. We revealed that the cumulative number of macrofungal species stabilized into distinct distribution patterns with localized hotspots of predicted macrofungal diversity with sampling efforts greater than approximately 30 years. Our research demonstrates the utility and importance of the long-term accumulated open biodiversity data in the study of macrofungal diversity and distribution at the national level.

### 4.1. The Importance of Long-Term Accumulation of Macrofungal Data

Given the ephemeral, cryptic, and climate-driven characteristics of macrofungal occurrence, there is no doubt that the study of macrofungal diversity and distribution patterns will require intensive and long-term sampling [14,21,22], which can be time consuming and expensive for field sampling on a large spatial extent [9,16]. Compared to systematic surveys, open biodiversity data are able to integrate information from multiple sources across space and time, though it can be heterogeneous in sampling approaches due to different study objectives and, therefore, may contain spatial or temporal bias [27,82]. However, these long-term accumulated biodiversity data from various sources do provide a wealth of species information. Our research found that 50 years of georeferenced open macrofungal data in Norway and Sweden collected nearly 3000 macrofungal species in each country. The sampling coverage spread across almost the whole countries (see Figure 2f), which showed the least sampling coverage, as we only considered the presence recording. Additionally, we found that the cumulative number of macrofungal species used in the models reached an asymptote following a long-term accumulation of data of more than 30 years in Norway and Sweden (Figure 3a).

These long-term accumulated open macrofungal data gathered large quantities of data, which might be scalable to the spatial and/or temporal extents required to understand species diversity and distribution patterns at a large spatial extent [83]. We predicted (see Figure 6) that the southern area of Norway and Sweden had the highest macrofungal diversity. A study using the catalogue data on functional groups, frequency of occurrence, geographic distribution, and habitat associations of 3196 macrofungal species in Sweden also found that there were particularly rich macrofungi in the southern boreo-nemoral and nemoral zones [84], which matches our predicted hotspots of macrofungi in Sweden based on accumulated GBIF data. The top 1% richness hotspot in Norway was also predicted using the GBIF-accumulated data to be in the boreo-nemoral zones, which matches the findings of Hagen et al. [85]. Nilsson et al. [86] and Yu et al. [50] confirmed that these boreo-nemoral and nemoral zones, which are dominated by either coniferous or broadleaf trees, provided ideal habitats for numerous fungi species and, in particular, macrofungi.

The importance of the long-term accumulation of open biodiversity data is also decided by the possible temporal and/or spatial bias among the datasets. As found in our research, there was a clear increase in sampling effort (both sampling time and spatial data coverage) of the GBIF macrofungal databases. However, the predicted highly similar and reasonable diversity pattern of macrofungal species showed up after accumulating data for about 30 years. Although the accumulation of macrofungal species we used in the model already reached an asymptote after about 20 years, the coverage of the area with macrofungal presence data is still limited. As shown in Figure 7, the mean SSIM index between 20 and 30 years is higher than 0.8; however, there are large amounts of patches with SSIM values smaller than 0.6. This result implies that the predicted diversity pattern based on data of 20 years still presented many detailed differences compared to the pattern predicted by 30 years’ data, meaning that there is no stabilized pattern across the whole country yet. The possible explanation is that there is limited spatial coverage during this period, which could cover a biased range of environmental factors (here, climatic factors) [36]. For example, as shown in Figure 2c, there are no occurrence data in the large area in the north part of the two countries; therefore, the climatic information inputted in the model might not cover, e.g., low temperature. Hence, there might be an uneven representation of climatic gradients by these occurrence records, and the model cannot predict accurate habitat suitability for macrofungi based on the unevenly represented predictor variables [87]. Nevertheless, the spatial coverage kept increasing with the long-term accumulation of data (see Figure 3b), and at least half of the total area was accumulated. The predicted pattern using more than 30 years’ data reached significantly high similarity, with small variations among patches. Thus, a relatively stabilized diversity pattern across the country was reached. Therefore, bias among the open macrofungal data could be alleviated by the long-term accumulation of data over a large spatial extent.

### 4.2. Uncertainty and Limitations

We note that our research included data collected over 50 years, and there may also be environmental, land-use, and climate change factors in this period. Thus, the information on the presence of macrofungi may contain macrofungal shifts affected by the environmental change or species adaptation to a changing environment. However, it is not possible to disentangle the possible effect or to conclude causal relationships between such diverse environmental changes and macrofungal diversity based solely on occurrence data [88,89]. Nevertheless, we were able to predict the relatively stabilized diversity and distribution patterns in the two countries after a long-term accumulation of data.

Norway and Sweden are among the regions where the fungi have been most thoroughly investigated [15,51]. However, we noted that only about half of the macrofungi species fulfilled the requirement of the species distribution model we applied, i.e., occupied at least 30 grid cells across the study area. The resolution of the grid cell we used is 25 km^2^, which is decided by the resolution of the recordings we downloaded. Thus, species with more than 30 grid cells could distribute in a large area, which means that the species have a high chance of being common species. Although the predicted macrofungal diversity and distribution patterns of these common species may not have significant differences with more relatively rare species added, there is still an urgent need for macrofungal occurrence data with higher resolution for a more accurate study of the fungi kingdom. In addition, there is an emerging tool for the study of fungi—environmental DNA (eDNA), which is thought to be a powerful means of biodiversity monitoring [90] and is able to detect more cryptic fungal species, especially those species with indistinguishable hyphae growing below ground [91,92,93]. However, like any other monitoring approach, eDNA can only detect a proportion of the total sites occupied by a given species [90]. Therefore, future studies combining the fruiting bodies and eDNA data may provide more comprehensive information on fungal occurrence, though how to combine data from these two methods needs further study.

Although the predicted results should be interpreted cautiously given the uncertainties, our research suggests that these long-term accumulated open-access datasets provide substantial species information, which can increase the capacity of researchers to study and understand the important role of macrofungi in regional and global ecosystem function. Nevertheless, the reliability of the open data or how long open macrofungal data need to be accumulated depends on the specific database in different regions, specific study objectives and specific methods for using these data [94]. Our research also suggests carefully exploring the temporal and spatial distribution of the database, as well as the accumulation of species numbers inside the study area along the accumulation of data, before using the long-term accumulated open macrofungal data.

## Figures and Tables

**Table 1:** The number of species and the number of grid cells in the original macrofungal database and the final macrofungal occurrence data used in our model for each sampling period and each country.

			10 Years Sampling	20 Years Sampling	30 Years Sampling	40 Years Sampling	50 Years Sampling
Number of species	Sweden	original macrofungi	1715	2243	2538	2783	2999
		macrofungi used in our model	330	775	1053	1243	1422
	Norway	original macrofungi	1717	2153	2420	2655	2901
		macrofungi used in our model	158	320	587	927	1168
Number of grid cells	Sweden	original macrofungi	1877	3900	7860	10,388	12,851
		macrofungi used in our model	1581	3678	7742	10,324	12,830
	Norway	original macrofungi	1800	2504	3774	5703	7167
		macrofungi used in our model	1296	2036	3515	5559	7100

**Table 2:** Bioclimatic variables used for modelling the diversity and distribution of macrofungi in this study.

Variables	Description	Unit
Bio01	Annual mean temperature	°C
Bio04	Temperature seasonality	%
Bio07	Temperature Annual Range	°C
Bio12	Annual precipitation	mm
Bio15	Precipitation seasonality	%
Dominantree	Dominant tree species	
Richness	Tree species richness	
Simpson’s Index	Diversity index of tree species	
pH	Soil pH in water	
SOC	Soil organic carbon content	g/kg
BD	Bulk density of the fine earth fraction	kg/dm^3^

## Data Availability

All datasets used are third-party datasets available freely on public repositories. The occurrence data for fungal species are freely available from the Global Biodiversity Information Facility. The bioclimatic variables (v.2.0) are available from the WorldClim database (http://www.worldclim.org/, accessed on 19 October 2021). The 20 tree species distribution maps are available from the European Forest Institute (https://www.efi.int/knowledge/maps/treespecies, accessed on 3 June 2021). The soil data are available from the SoilGrid database (https://files.isric.org/soilgrids/latest/data/, accessed on 2 September 2022). The original fungal occurrence data used in this study are available at: https://doi.org/10.15468/dl.ejuvj6 (accessed on 17 February 2022), https://doi.org/10.15468/dl.8x7f2z (accessed on 17 February 2022), and https://doi.org/10.15468/dl.tknmhk (accessed on 17 February 2022); and all input files to reproduce our models are available in Dryad Digital Repository at https://doi.org/10.5061/dryad.1ns1rn8x7 (accessed on 17 February 2022).
